# Screening and identification of critical biomarkers in erectile dysfunction: evidence from bioinformatic analysis

**DOI:** 10.7717/peerj.8653

**Published:** 2020-02-28

**Authors:** Jialiang Hui, Ruiyu Liu, Haibo Zhang, Shuhua He, Anyang Wei

**Affiliations:** Department of Urology, Nanfang Hospital, Southern Medical University, Guangzhou, Guangdong, China

**Keywords:** Erectile dysfunction, ED, Biomarkers, Bioinformatics

## Abstract

**Purpose:**

Erectile dysfunction (ED) is one of the most common male-disease globally. Despite efforts to explain its pathogenesis, the molecular mechanisms of ED are still not well understood.

**Methods:**

The microarray dataset GSE10804 was downloaded from the Gene Expression Omnibus (GEO) to find candidate genes in ED progression. After differentially expressed genes (DEGs) were identified, functional enrichment analysis was performed. In addition, a protein-protein interaction network (PPI) was established and module analysis was performed through the STRING and Cytoscape.

**Results and Conclusions:**

A total of 618 DEGs were identified in all, containing 430 downregulated genes and 188 upregulated genes. The enriched functions and pathways of the DEGs include transcription from RNA polymerase II promoter, cell adhesion, calcium ion binding, receptor binding, Akt signaling pathway, receptor interaction, protein digestion, and absorption. We picked out twenty-five hub genes, with biological process (BP) analyses revealing that the genes were principally associated with cellular responses to amino acid stimuli, extracellular matrix structural constituent, collagen trimer, protein digestion and absorption, ECM-receptor interaction and PI3K-Akt signaling pathway. To sum up, DEGs and hub genes distinguished in this study not only help us understand the molecular mechanisms behind the carcinogenesis and progression of ED, but also play a part in the diagnosis and treatment of ED by providing candidate targets.

## Introduction

Erectile dysfunction is a medical condition in which a man is unable to maintain an erection for vaginal intercourse (1993). Actually, a description of poor penile erection was firstly mentioned as early as 5,000 years ago in an ancient Egyptian literature (Shamloul & Ghanem, 2013). Erectile dysfunction is known to increase with age. In a cross-sectional study based on community, the prevalence of this condition, in men in the 40–49 age group, was about 5% for severe dysfunction (Feldman et al., 1994). For men in the 70–79 age group, the prevalence rates were 15% and 34%, for complete or severe dysfunction, respectively. By 2025, it is speculated that the global prevalence of ED will reach up to 322 million cases (Bacon et al., 2003). Men aged older than 40 years are more susceptible to erectile dysfunction. Several other medical diseases are linked to erectile dysfunction such as diabetes mellitus (Brown et al., 2005), tobacco use, central neuropathologic conditions, cardiovascular disease (Billups, 2005), lower urinary tract symptoms of benign prostatic hyperplasia (McVary, 2005), and metabolic syndrome (Kupelian et al., 2006).

The vessels of the vascular system (and lymphatics) contain a single-cell-layered lining called the endothelium. Based on its uniqueness and functions, the endothelium is thought to be an organ (Aird, 2004). The parts of the endothelium are diverse regarding biochemical properties, functional characteristics, and cell surface markers (Ho et al., 2003; Kallmann et al., 2002). The understanding of the heterogeneity of this factor in the endothelial cells (ECs) mainly originates from several examinations of various body tissues and organ specimen by microarray analysis (Chi et al., 2003; Ho et al., 2003; Kallmann et al., 2002; Podgrabinska et al., 2002).

Endothelial dysfunction is considered as the genesis of ED, especially that of the systemic vasculature (Gerber et al., 2015). Plenty of studies have been carried out to investigate the function of the endothelium both during physiological erection and also in the ED state (Hurt et al., 2002). The endothelium not only functions as a passive barrier but also as a critical regulator of muscle tone and vascular circulation, which are coordinated by signals originating from mechanical stimuli, humoral and neural sources.

In order to facilitate research into the progression of ED in terms of the functional pathways and differentially expressed genes (DEGs), many technologies such as bioinformatics analysis and microarray technology have been widely used. However, because it is exceedingly difficult to obtain penile tissue specimens from patients with ED, there are few studies of human tissue. Thus, here, the DEGs between non-ED tissues and ED tissues were searched and obtained by screening the Gene Expression Omnibus (GEO) for mRNA microarray datasets. Subsequently, protein-protein interaction (PPI) networks, pathway enrichment analyses of the Kyoto Encyclopedia of Genes and Genomes (KEGG) and Gene Ontology (GO), were performed to explore the mechanisms that underlie the pathogenesis of ED. In conclusion, we identified 618 DEGs and 25 hub genes as possible biological markers for ED.

## Materials & Methods

### Microarray data

GEO (http://www.ncbi.nlm.nih.gov/geo) (Barrett et al., 2013; Edgar, Domrachev & Lash, 2002), a public functional genomics data repository, provides chips, microarrays, and high throughput gene expression data. Three gene expression datasets GSE10804 (Wessells et al., 2009) were downloaded from GEO (Affymetrix GPL571platform, Affymetrix Human Genome U133A 2.0 Array). The probes were transformed into the corresponding gene symbols on the basis of annotation information on the platform. The GSE10804 datasets had 3 groups: 5 samples are cultured human corpus cavernosum endothelial cells (HCCEC) from a donor with erectile dysfunction; 3 samples are cultured human umbilical vein endothelial cells (HUVEC) from a donor without erectile dysfunction, and 4 samples are cultured human coronary artery endothelial cells (HCAEC) from a donor without erectile dysfunction. We have standardized that the 5 ED samples compared to the 7 non-ED samples as a whole.

### Identification of DEGs

The DEGs in the ED and non-ED specimen were picked out from GEO2R (http://www.ncbi.nlm.nih.gov/geo/geo2r), which is a platform for examing DEGs across experimental conditions by comparing multiple datasets in a GEO series. The genes with multiple probes were averaged, and the probes that lacked gene symbols were removed. *P*-value <0.01 and logFC (fold change) >1 represented statistical significance.

### Analysis of GO enrichment and KEGG networks for DEGs

The Database for Annotation, Visualization and Integrated Discovery (DAVID; http://david.ncifcrf.gov) (version 6.8) (Huang da, Sherman & Lempicki, 2009) offers an analytical platform with a comprehensive source of annotated information of proteins and genes which can be extracted and analyzed. On the other hand, KEGG (Kyoto Encyclopedia of Genes and Genomes) is a capable platform that is a knowledge base for systematic analysis of gene functions, linking genomic information with higher-order functional information (Kanehisa & Goto, 2000; Kanehisa et al., 2019; Kanehisa, 2019). GO, a significant bioinformatics tool enables us to annotate genes according to biological processes (Gene Ontology, 2015). In order to study the functions of DEGs, DAVID online database was used to analyze the biological. *P* < 0.01 was taken to represent statistical significance. ImageGP (http://www.ehbio.com/ImageGP/) is used to draw Enrichment Plot for GO and KEGG.

### Construction of PPI networks and module analyses

In this study, the STRING database (STRING; http://string-db.org) (version 10.0) (Szklarczyk et al., 2017) was taken to predict the PPI network and to determine protein-protein interactions and to investigate the molecular basis of diseases. The interactions with an average score of greater than 0.4 were chosen a the cut-off for statistical significance. To visualize molecular interaction networks, the Cytoscape (version 3.4.0), a bioinformatics software, is often utilized (Saito et al., 2012). This software contains an APP known as The plug-in Molecular Complex Detection (MCODE) (version 1.4.2), which is used to cluster-specific networks according to the on the topology to reveal the regions that are densely connected (Saito et al., 2012). Cytoscape was used to draw the PPI networks, and the MCODE helped to identify the most remarkable module in the PPI networks. The selecting criteria was: *k*-score = 2, Max depth = 100, node score cut-off = 0.2, degree cut-off = 2, and MCODE scores >5. Then, the DAVID tool was applied to analyze the GO and KEGG genes in the module.

### Selection and analysis of Hub genes

Hub genes with a degree greater than or equal to 10 were selected. Analysis and visualization of the hub genes were conducted by the Biological Networks Gene Oncology tool (BiNGO) (version 3.0.3) plugin of Cytoscape (Saito et al., 2012).

## Results

### Identification of DEGs in the ED

**Figure 1:**
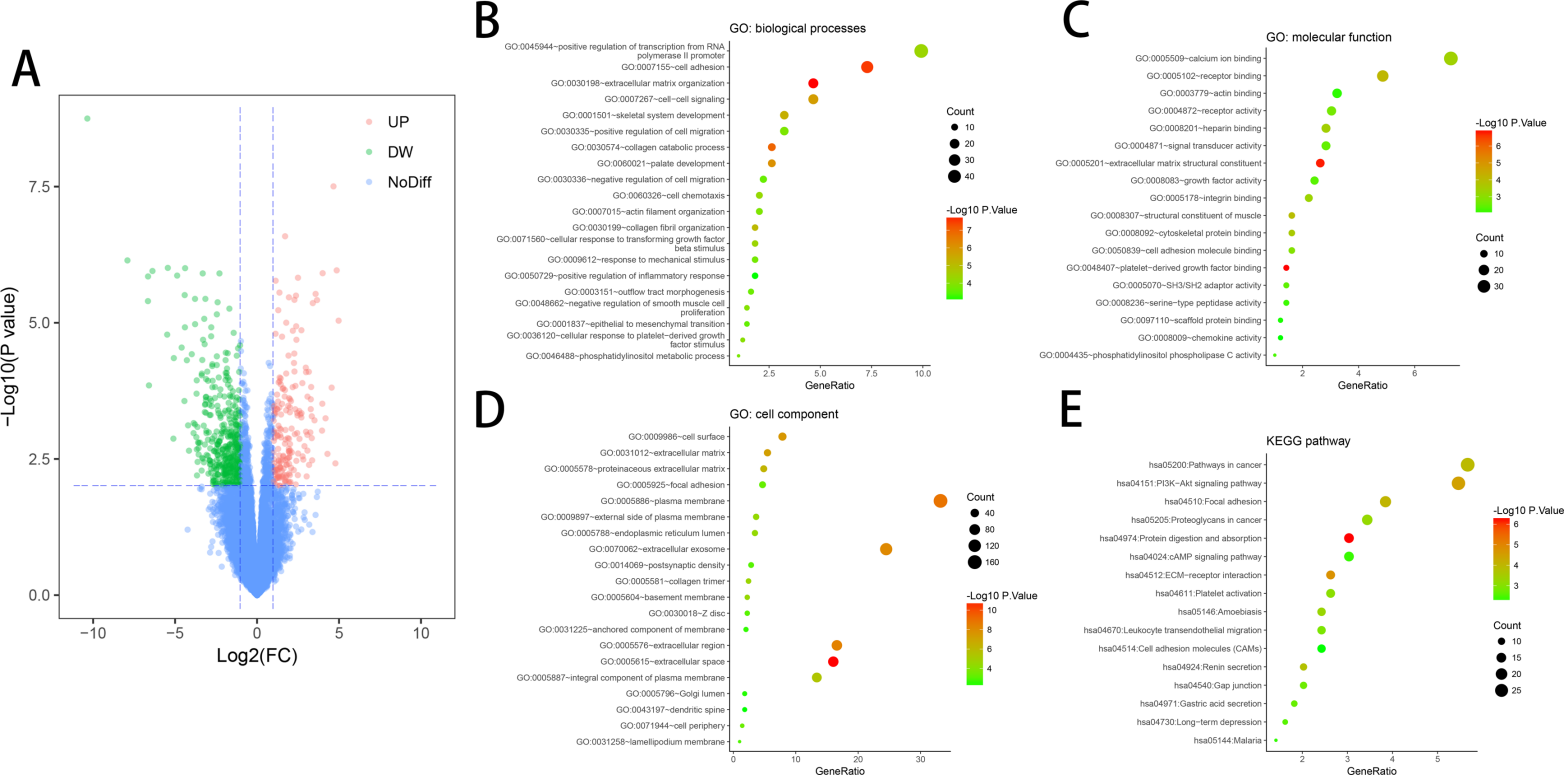
DEGs in ED and GO & KEGG pathway enrichment analysis of DEGs. (A) DEGs were selected with a fold change >1 and *P*-value < 0.01 among the mRNA expression profiling set GSE10804. (B) Biological processes (BP) of DEGs. (C) Molecular function (MF) of DEGs. (D) Cell component (CC) of DEGs. (E) KEGG pathway of DEGs.

DEGs (618 in GSE10804) were identified (Fig. 1A) after standardizing the microarray data. The dataset consisted of 430 downregulated genes and 188 upregulated genes between ED endothelial cells and non-ED endothelial cells.

### KEGG and GO enrichment analyses of DEGs

DAVID tool is used for the analysis of DEGs classification, functional and pathway enrichment. Results of GO analyses revealed that changes in BP of DEGs had great enrichment in increasing gene expression from RNA polymerase II promoter, cell adhesion, extracellular matrix organization, cell–cell signaling and positive control of cell migration (Fig. 1B). Molecular function (MF) mainly had changes in calcium-binding, receptor binding, sequence-specific DNA binding, actin binding and receptor activity (Fig. 1C). Cell component (CC) of DEGs primarily had changes in the cell surface, proteinaceous extracellular matrix, focal adhesion and plasma membrane (Fig. 1D). KEGG pathway analyses showed that DEGs mainly had enrichment in Focal adhesion, the Akt signaling pathway, receptor interaction, Protein digestion and absorption, and Pathways in cancer (Fig. 1E).

Functional and pathway enrichment analyses were conducted on downregulated and upregulated DEGs respectively for further analyses of the biological classification. In the upregulated DEGs, the Results of GO analyses demonstrated that the gamma-aminobutyric acid signaling pathway and osteoclast differentiation had changes in BP (Table 1). KEGG analysis showed the main enrichment in the Renin secretion of downregulated DEGs (Table 1). MF and CC showed no results in the upregulated DEGs. In the downregulated DEGs, the Results of GO analyses showed that the remarkable enhancement of changes in BP was in the collagen catabolic process, extracellular matrix organization, and cell adhesion (Table 2). Changes in MF were greatly improved in calcium ion binding, platelet-derived growth factor binding, and heparin-binding (Table 2). Changes in CC primarily emerged in the extracellular matrix, extracellular region and extracellular matrix (Table 2). KEGG pathway analysis revealed that the downregulated DEGs were mainly enhanced in the PI3K-Akt signaling pathway, ECM-receptor interaction, and Focal adhesion (Table 2).

**Table 1:** GO and KEGG pathway enhancement analysis of upregulated DEGs in ED samples.

**Term**	**Description**	**Count in gene set**	**Gene ratio**	***P*-value**
**GOTERM_BP_DIRECT**				
GO:0007214	Gamma-aminobutyric acid signaling pathway	3	1.923	0.006
GO:0030316	Osteoclast differentiation	3	1.923	0.009
**KEGG_PATHWAY**				
ptr04924	Renin secretion	5	3.205	0.004

**Notes.**

Abbreviations GO Gene Ontology KEGG Kyoto Encyclopedia of Genes and Genomes DEGs differentially expressed genes ED erectile dysfunction

**Table 2:** GO and KEGG pathway enrichment analysis of downregulated DEGs in ED samples.

**Term**	**Description**	**Count in gene set**	**Gene ratio**	***P*-value**
**GOTERM_BP_DIRECT**				
GO:0030198	Extracellular matrix organization	22	6.452	1.28E−10
GO:0007155	Cell adhesion	30	8.798	1.20E−08
GO:0030574	Collagen catabolic process	12	3.519	2.61E−08
GO:0060021	Palate development	12	3.519	1.65E−07
GO:0001501	Skeletal system development	15	4.399	2.98E−07
GO:0030199	Collagen fibril organization	9	2.639	5.20E−07
GO:0007267	Cell–cell signaling	18	5.279	6.48E−06
GO:0060326	Cell chemotaxis	9	2.639	2.80E−05
GO:0046488	Phosphatidylinositol metabolic process	5	1.466	3.61E−05
GO:0030335	Positive regulation of cell migration	13	3.812	1.90E−04
**GOTERM_MF_DIRECT**				
GO:0005201	Extracellular matrix structural constituent	13	3.812	2.69E−09
GO:0048407	Platelet-derived growth factor binding	7	2.053	1.50E−08
GO:0005509	Calcium ion binding	29	8.504	1.32E−04
GO:0008201	Heparin binding	12	3.519	1.74E−04
GO:0008092	Cytoskeletal protein binding	7	2.053	2.26E−04
GO:0005178	Integrin binding	9	2.639	6.76E−04
GO:0003779	Actin binding	14	4.106	1.85E−03
GO:0008009	Chemokine activity	6	1.76	1.94E−03
GO:0008083	Growth factor activity	10	2.933	2.95E−03
GO:0005518	Collagen binding	6	1.76	4.71E−03
**GOTERM_CC_DIRECT**				
GO:0005615	Extracellular space	64	18.768	1.48E−12
GO:0005576	Extracellular region	68	19.941	4.84E−11
GO:0031012	Extracellular matrix	26	7.625	1.53E−10
GO:0005578	Proteinaceous extracellular matrix	21	6.158	8.74E−08
GO:0009986	Cell surface	30	8.798	1.74E−07
GO:0070062	Extracellular exosome	87	25.513	2.27E−07
GO:0005788	Endoplasmic reticulum lumen	15	4.399	1.01E−05
GO:0005604	Basement membrane	10	2.933	1.18E−05
GO:0005886	Plasma membrane	107	31.378	2.58E−05
GO:0009897	External side of plasma membrane	15	4.399	3.23E−05
**KEGG_PATHWAY**				
hsa04512	ECM-receptor interaction	13	3.812	3.56E−07
hsa04151	PI3K-Akt signaling pathway	24	7.038	1.53E−06
hsa04510	Focal adhesion	18	5.279	2.36E−06
hsa04974	Protein digestion and absorption	12	3.519	3.03E−06
hsa05200	Pathways in cancer	23	6.745	4.27E−05
hsa05146	Amoebiasis	11	3.226	1.03E−04
hsa05205	Proteoglycans in cancer	14	4.106	4.32E−04
hsa04540	Gap junction	9	2.639	6.61E−04
hsa04670	Leukocyte transendothelial migration	10	2.933	1.10E−03
hsa04730	Long-term depression	7	2.053	1.93E−03

**Notes.**

Abbreviations GO Gene Ontology KEGG Kyoto Encyclopedia of Genes and Genomes DEGs differentially expressed genes ED erectile dysfunction

### Construction of PPI networks and module analyses

**Figure 2:**
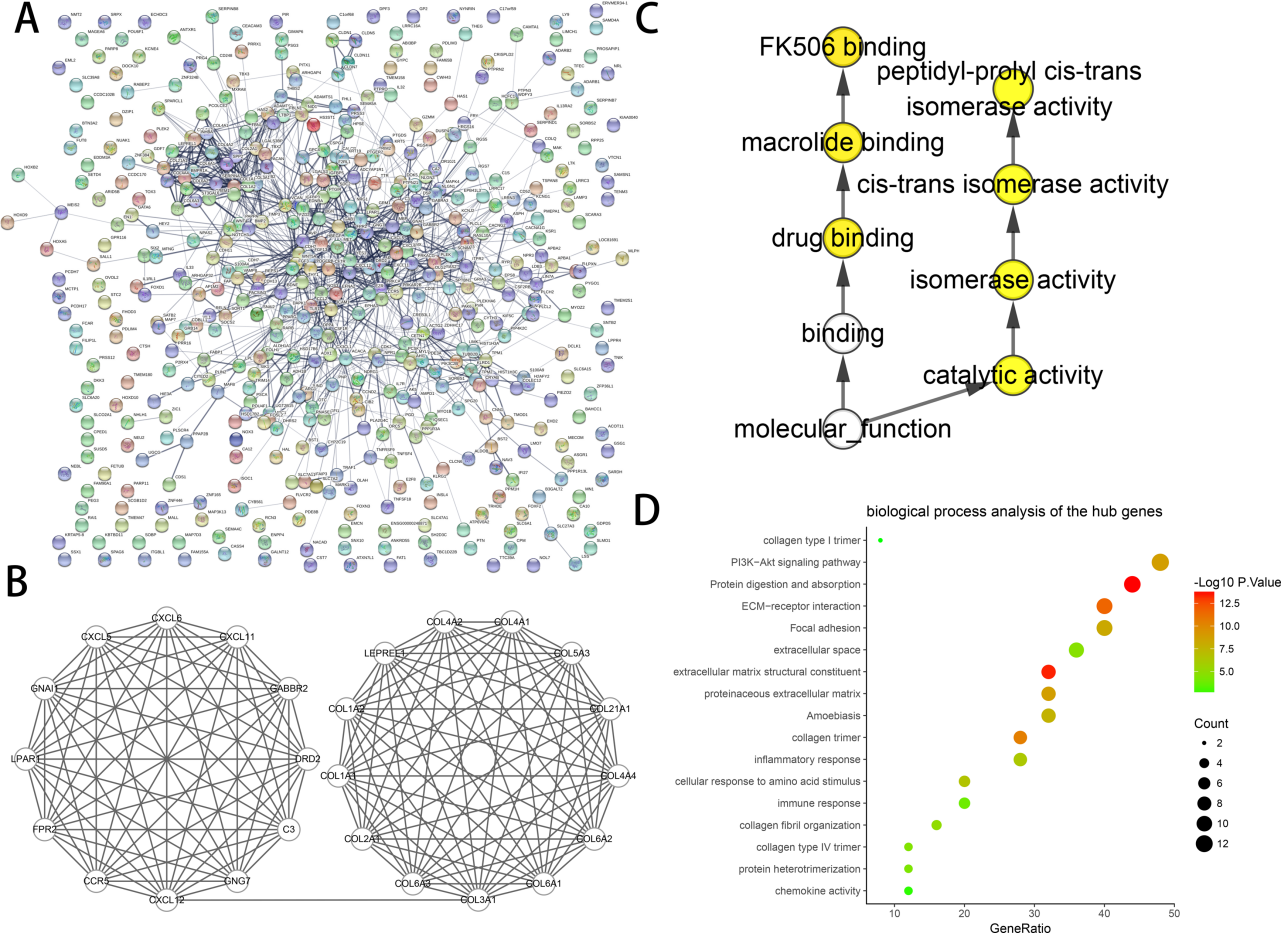
PPI network and the most significant module of DEGs. (A) The PPI network of DEGs was created using Cytoscape. (B) The most critical module was obtained from the PPI network with 25 nodes and 145 edges. (C) The biological process analysis of hub genes was constructed using BiNGO. *P* < 0.01 was considered statistically significant. (D) The biological process analysis of hub genes was created using STRING.

The constructed PPI networks of DEGs are shown in (Fig. 2A), and the most significant module was obtained using Cytoscape (Fig. 2B). The DAVID tools were applied in the functional analyses of genes associated with this module. We found that the genes primarily had enrichment in cell responses to amino acid stimuli, extracellular matrix structural constituent and collagen trimer (Table 3).

### Selection and analysis Hub gene

**Figure 3:**
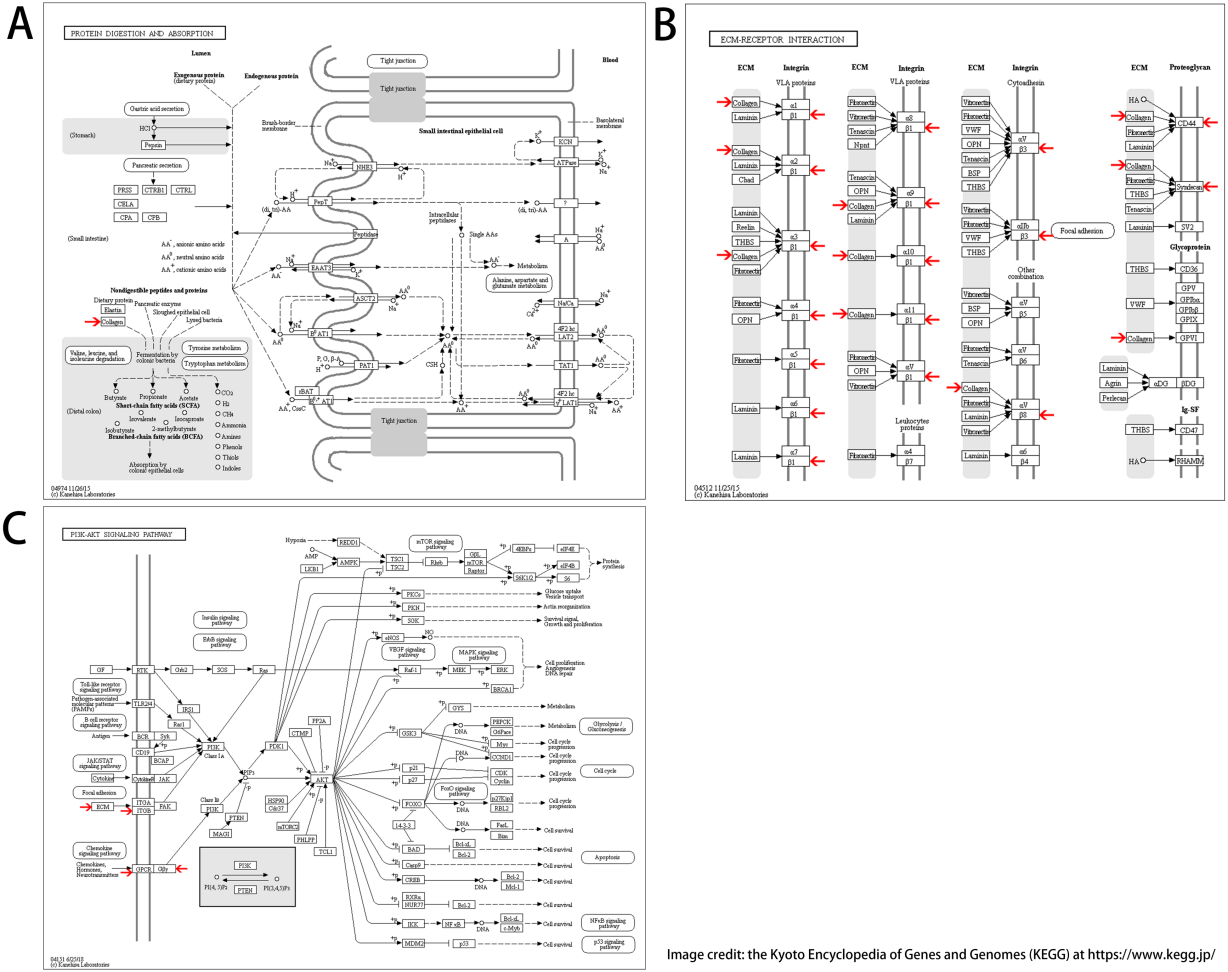
KEGG pathway enrichment analysis of hub DEGs. (A) Protein digestion and absorption. (B) ECM-receptor interaction. (C) PI3K-Akt signaling pathway.

We found 25 hub genes with degrees ≥10. Table 4 shows the list of the functions, abbreviations, and names of the genes. The analysis of the biological processes of the 25 genes is shown in Figs. 2C and 2D. For the determination of the categorization of the hub DEGs, the DAVID tool was used. Results of GO analyses demonstrated that changes in BP of hub DEGs remarkably emerged in cell responses to amino acids stimuli, and inflammatory response (Table 3, Figs. 2C and 2D) Changes in molecular function (MF) primarily enhanced in extracellular matrix structural constituent and chemokine activity (Table 3, Figs. 2C and 2D). Changes in cell component (CC) mainly increased in the collagen trimer and proteinaceous extracellular matrix (Table 3, Figs. 2C and 2D). Based on the analysis of the KEGG pathway, we found that the enrichment of hub DEGs was mainly in Protein digestion and absorption (Fig. 3A), ECM-receptor interaction (Fig. 3B) and PI3K-Akt signaling pathway (Table 3, Figs. 2C, 2D and 3C).

**Table 3:** GO and KEGG pathway enrichment analysis of hub DEGs in ED samples.

**Term**	**Description**	**Count in gene set**	**Gene ratio**	***P*-value**
**GOTERM_BP_DIRECT**				
GO:0071230	Cellular response to amino acid stimulus	5	20	2.53E−07
GO:0006954	Inflammatory response	7	28	4.93E−07
GO:0030199	Collagen fibril organization	4	16	1.06E−05
GO:0070208	Protein heterotrimerization	3	12	4.91E−05
GO:0006955	Immune response	5	20	1.44E−04
**GOTERM_MF_DIRECT**				
GO:0005201	Extracellular matrix structural constituent	8	32	4.34E−14
GO:0008009	Chemokine activity	3	12	1.84E−03
**GOTERM_CC_DIRECT**				
GO:0005581	Collagen trimer	7	28	3.48E−11
GO:0005578	Proteinaceous extracellular matrix	8	32	1.54E−09
GO:0005615	Extracellular space	9	36	2.31E−05
GO:0005587	Collagen type IV trimer	3	12	3.31E−05
GO:0005584	Collagen type I trimer	2	8	3.04E−03
**KEGG_PATHWAY**				
ptr04974	Protein digestion and absorption	11	44	2.40E−14
ptr04512	ECM-receptor interaction	10	40	2.56E−12
ptr04151	PI3K-Akt signaling pathway	12	48	1.40E−09
ptr04510	Focal adhesion	10	40	5.78E−09
ptr05146	Amoebiasis	8	32	2.31E−08

**Notes.**

Abbreviations GO Gene Ontology KEGG Kyoto Encyclopedia of Genes and Genomes DEGs differentially expressed genes ED erectile dysfunction

**Table 4:** Functional roles of 25 hub genes with degree ≥10.

**Gene symbol**	**Full name**	***P*-Value**	**logFC**
C3	Complement component 3	3.36E−03	1.324
CCR5	C-C motif chemokine receptor 5	6.96E−03	−1.395
COL1A1	Collagen type I alpha 1 chain	1.78E−09	−10.353
COL1A2	Collagen type I alpha 2 chain	1.36E−06	−4.873
COL21A1	Collagen type XXI alpha 1 chain	9.97E−03	−1.804
COL2A1	Collagen type II alpha 1 chain	4.85E−05	−1.92
COL3A1	Collagen type III alpha 1 chain	1.16E−04	−2.991
COL4A1	Collagen type IV alpha 1 chain	6.43E−03	−1.865
COL4A2	Collagen type IV alpha 2 chain	1.85E−03	−1.871
COL4A4	Collagen type IV alpha 4 chain	5.07E−03	−1.064
COL5A3	Collagen type V alpha 3 chain	9.11E−03	−2.508
COL6A1	Collagen type VI alpha 1 chain	4.87E−05	−4.282
COL6A2	Collagen type VI alpha 2 chain	1.65E−05	−5.482
COL6A3	Collagen type VI alpha 3 chain	7.16E−07	−7.906
CXCL11	C-X-C motif chemokine ligand 11	2.30E−03	−1.368
CXCL12	C-X-C motif chemokine ligand 12	4.03E−03	−2.309
CXCL5	C-X-C motif chemokine ligand 5	1.29E−03	−2.202
CXCL6	C-X-C motif chemokine ligand 6	4.23E−03	−1.948
DRD2	Dopamine receptor D2	1.71E−03	−1.038
FPR2	Formyl peptide receptor 2	9.28E−04	1.731
GABBR2	Gamma-aminobutyric acid type B receptor subunit 2	3.17E−06	2.374
GNAI1	G protein subunit alpha i1	1.73E−04	−1.107
GNG7	G protein subunit gamma 7	4.59E−03	−1.475
LEPREL1	P3H2 (LEPREL1) prolyl 3-hydroxylase 2	–	–
LPAR1	Lysophosphatidic acid receptor 1	2.28E−03	−2.632

## Discussion

Men aged older than 40 years are more susceptible to erectile dysfunction. Several other medical diseases are linked to erectile dysfunction such as diabetes mellitus (Brown et al., 2005), tobacco use, central neuropathologic conditions (e.g., hemorrhagic or ischemic stroke and Parkinson’s disease), cardiovascular disease (Billups, 2005), lower urinary tract symptoms of benign prostatic hyperplasia (McVary, 2005), metabolic syndrome (Kupelian et al., 2006). More than 70% of coronary artery disease patients present with moderate to severe erectile dysfunction which might occurred before cardiovascular events. Moreover, endothelial dysfunction is also linked with atherosclerosis (Billups, 2005). The likelihood of developing erectile dysfunction tends to increase with prolonged diabetes probably due to a high level of glycated hemoglobin. Some drugs are also suspected to cause erectile dysfunction based on a clinical diagnosis of 25% of the patients diagnosed with erectile dysfunction (Thomas et al., 2003). However, little is understood about the molecular mechanism of ED at present. The NO-cGMP signaling pathway has been shown to be critical in the pathogenesis of ED (Garcia-Cardoso et al., 2010). These results have also been applied in clinical practice, as selective inhibitors of 5-type phosphodiesterase specific to cGMP, such as sildenafil and tadalafil have achieved significant clinical effects in the treatment of ED. RhoA/Rho-kinase contributes to the development and progression of ED. It has been demonstrated that the vasodilation of NO may be due to the reduction of calcium levels by NO-cGMP-PKG on one hand, and the relaxation effect by blocking the RhoA/Rho-kinase calcium-sensitive pathway on the other hand (Jin et al., 2006). Recently, it has also been found that the MAPK signaling pathway was demonstrated to affect the NO-cGMP signaling pathway by regulating related kinases, which indirectly leads to the occurrence of ED. The primary treatments for erectile dysfunction are oral PDE5-Is. Other interventions are psychotherapy, penile devices, testosterone therapy, injection therapies, and psychotherapy, etc (1993). But there is no curable medications for ED that can be completely cured, which may be one of the reasons for the poor patient prognosis. Therefore, potential markers for diagnosis and treatment with high efficiency are urgently demanded. Microarray technology allows us to explore the genetic alterations in the ED and there is evidence surporting it to be a useful method for identifying new biomarkers in other diseases. An article conducted a large-scale genome-wide association study of erectile dysfunction in the multiethnic Kaiser Permanente Northern California Genetic Epidemiology Research in Adult Health and Aging cohort and UK Biobank. They find the single-minded family basic helix-loop-helix transcription factor 1 (SIM1) gene is a mechanism that is specific to sexual function (Jorgenson et al., 2018).

Here, we analyzed the mRNA microarray datasets to identify DEGs in ED cells and non-ED cells. 618 DEGs were obtained in all datasets, including 430 downregulated genes and 188 upregulated genes. The reciprocities among the DEGs were searched out by KEGG enrichment analyses. The DEGs largely had enrichment in positively regulating transcription from RNA polymerase II promoter, cell adhesion, calcium ion binding, receptor binding, Akt signaling pathway, receptor interaction, Protein digestion, and absorption. Lianjun Pan and Jiehua Ma speculated that many lncRNAs are involved in the regulation of muscle contraction and relaxation through ion channel activity. They suggested that a muscle contraction disorder induced by abnormal ion channel activity, collagen deposition and fibrosis might play a critical role in the pathogenesis of ED (Pan et al., 2015). Changes to the endothelial cell–cell junction integrity like in the case of increases in vascular permeability and decreased cell–cell junction proteins were observed in the cavernous tissue of the diabetic mouse. The presence of hollow junctions in diabetes state may explain the high prevalence of erectile dysfunction that occurs before other cardiovascular conditions (Ryu et al., 2013). Large conductance, voltage, and Ca2+ activated K+ channels are widely present in many cells of the body, which play a part in regulating the neuronal excitability and smooth muscle tone. When their function is compromised, they may bring about the development of erectile dysfunction, hypertension and overactive bladder (Carmona et al., 2016). Akt plays an essential role in regulating cell homeostasis through its effects on several downstream effectors (Carmona et al., 2016). Prior studies have shown that phosphorylation of eNOS by Akt causes prolonged NO production and maximal construction and influences penile erection (Zhang et al., 2011). Similarly, it was found that PI3K/Akt-eNOS activation mediated the effects of the A2B adenosine receptor on penile erection (Ryu et al., 2013).

We selected 25 DEGs as hub genes with difference degrees ≥10. Among these hub genes, COL3A1 showed the highest node degrees with 13. And other hub genes also confirmed the top node degrees with 12 (COL1A1, COL1A2, COL21A1, COL2A1, COL4A1, COL4A2, COL4A4, COL5A3, COL6A1, COL6A2, COL6A3, CXCL12, LEPREL1) and 11 (C3, CCR5, CXCL11, CXCL5, CXCL6, DRD2, FPR2, GABBR2, GNAI1, GNG7, LPAR1). A study identification of oxidative stress-induced gene expression profiles in cavernosal endothelial cells, cxcl12 showed a corresponding variation in the CECs and ED rat model compared with the results of the gene microarray analysis (Hu et al., 2015). While ORP in NHPs produced persistent erectile and urinary tract dysfunction. Periurethral injection of CXCL-12 was feasible and improved both urinary incontinence and erectile dysfunction and suggests (Zambon et al., 2018). A study about five immune agents (IgG, IgM, IgA, C4, and C3) was collected on the basis of the Fangchenggang Area Male Health and Examination Survey (FAMHES)and methods of traditional cross-sectional analysis were used. However, there was no clear relationship between ED risk in the baseline analyses and the tested indexes (C3: *P* = 0.737) (Chen et al., 2014). Another study on sexual dysfunction in male schizophrenia showed that correlation between two polymorphisms in the genes for the DRD2 and scores on questionnaires for erectile and sexual dysfunction were studied. The functional −141Ins/Del promoter region polymorphism of DRD2 was remarkably correlated with sexual dysfunction (Zhang et al., 2011). The remaining genes did not retrieve research literature related to erectile dysfunction.

## Conclusions

In conclusion, 618 DEGs and 25 hub genes with the possibility to be considered as diagnostic biomarkers for ED were identified However, additional investigations should be carried out to explore the biological function of these genes in the ED.

## Additional Information and Declarations

### Data Availability

The following information was supplied regarding data availability: Raw data is available at NCBI GEO: GSE10804.

## References

[1993] (1993). Consensus development conference statement. National Institutes of Health. Impotence. December 7–9, 1992. International Journal of Impotence Research.

[ref-2] Aird WC (2004). Endothelium as an organ system. Critical Care Medicine.

[ref-3] Bacon CG, Mittleman MA, Kawachi I, Giovannucci E, Glasser DB, Rimm EB (2003). Sexual function in men older than 50 years of age: results from the health professionals follow-up study. Annals of Internal Medicine.

[ref-4] Barrett T, Wilhite SE, Ledoux P, Evangelista C, Kim IF, Tomashevsky M, Marshall KA, Phillippy KH, Sherman PM, Holko M, Yefanov A, Lee H, Zhang N, Robertson CL, Serova N, Davis S, Soboleva A (2013). NCBI GEO: archive for functional genomics data sets–update. Nucleic Acids Research.

[ref-5] Billups KL (2005). Sexual dysfunction and cardiovascular disease: integrative concepts and strategies. American Journal of Cardiology.

[ref-6] Brown JS, Wessells H, Chancellor MB, Howards SS, Stamm WE, Stapleton AE, Steers WD, Van Den Eeden SK, McVary KT (2005). Urologic complications of diabetes. Diabetes Care.

[ref-7] Carmona FJ, Montemurro F, Kannan S, Rossi V, Verma C, Baselga J, Scaltriti M (2016). AKT signaling in ERBB2-amplified breast cancer. Pharmacology and Therapeutics.

[ref-8] Chen Y, Xin X, Zhang H, Xu J, Gao Y, Tan A, Yang X, Qin X, Hu Y, Mo Z (2014). Immunization associated with erectile dysfunction based on cross-sectional and genetic analyses. PLOS ONE.

[ref-9] Chi JT, Chang HY, Haraldsen G, Jahnsen FL, Troyanskaya OG, Chang DS, Wang Z, Rockson SG, Van de Rijn M, Botstein D, Brown PO (2003). Endothelial cell diversity revealed by global expression profiling. Proceedings of the National Academy of Sciences of the United States of America.

[ref-10] Edgar R, Domrachev M, Lash AE (2002). Gene Expression Omnibus: NCBI gene expression and hybridization array data repository. Nucleic Acids Research.

[ref-11] Feldman HA, Goldstein I, Hatzichristou DG, Krane RJ, McKinlay JB (1994). Impotence and its medical and psychosocial correlates: results of the Massachusetts Male Aging Study. Journal d Urologie.

[ref-12] Garcia-Cardoso J, Vela R, Mahillo E, Mateos-Caceres PJ, Modrego J, Macaya C, Lopez-Farre AJ (2010). Increased cyclic guanosine monophosphate production and endothelial nitric oxide synthase level in mononuclear cells from sildenafil citrate-treated patients with erectile dysfunction. International Journal of Impotence Research.

[ref-13] Gene Ontology C (2015). Gene Ontology Consortium: going forward. Nucleic Acids Research.

[ref-14] Gerber RE, Vita JA, Ganz P, Wager CG, Araujo AB, Rosen RC, Kupelian V (2015). Association of peripheral microvascular dysfunction and erectile dysfunction. Journal d Urologie.

[ref-15] Ho M, Yang E, Matcuk G, Deng D, Sampas N, Tsalenko A, Tabibiazar R, Zhang Y, Chen M, Talbi S, Ho YD, Wang J, Tsao PS, Ben-Dor A, Yakhini Z, Bruhn L, Quertermous T (2003). Identification of endothelial cell genes by combined database mining and microarray analysis. Physiological Genomics.

[ref-16] Hu C, Dong YY, Dong YH, Cui JF, Dai JC (2015). Identification of oxidative stress-induced gene expression profiles in cavernosal endothelial cells. Molecular Medicine Reports.

[ref-17] Huang da W, Sherman BT, Lempicki RA (2009). Systematic and integrative analysis of large gene lists using DAVID bioinformatics resources. Nature Protocols.

[ref-18] Hurt KJ, Musicki B, Palese MA, Crone JK, Becker RE, Moriarity JL, Snyder SH, Burnett AL (2002). Akt-dependent phosphorylation of endothelial nitric-oxide synthase mediates penile erection. Proceedings of the National Academy of Sciences of the United States of America.

[ref-19] Jin L, Liu T, Lagoda GA, Champion HC, Bivalacqua TJ, Burnett AL (2006). Elevated RhoA/Rho-kinase activity in the aged rat penis: mechanism for age-associated erectile dysfunction. FASEB Journal.

[ref-20] Jorgenson E, Matharu N, Palmer MR, Yin J, Shan J, Hoffmann TJ, Thai KK, Zhou X, Hotaling JM, Jarvik GP, Ahituv N, Wessells H, Van Den Eeden SK (2018). Genetic variation in the locus is associated with erectile dysfunction. Proceedings of the National Academy of Sciences of the United States of America.

[ref-21] Kallmann BA, Wagner S, Hummel V, Buttmann M, Bayas A, Tonn JC, Rieckmann P (2002). Characteristic gene expression profile of primary human cerebral endothelial cells. FASEB Journal.

[ref-22] Kanehisa M (2019). Toward understanding the origin and evolution of cellular organisms. Protein Science.

[ref-23] Kanehisa M, Goto S (2000). KEGG: kyoto encyclopedia of genes and genomes. Nucleic Acids Research.

[ref-24] Kanehisa M, Sato Y, Furumichi M, Morishima K, Tanabe M (2019). New approach for understanding genome variations in KEGG. Nucleic Acids Research.

[ref-25] Kupelian V, Shabsigh R, Araujo AB, O’Donnell AB, McKinlay JB (2006). Erectile dysfunction as a predictor of the metabolic syndrome in aging men: results from the Massachusetts Male Aging Study. Journal d Urologie.

[ref-26] McVary KT (2005). Erectile dysfunction and lower urinary tract symptoms secondary to BPH. European Urology.

[ref-27] Pan L, Ma J, Pan F, Zhao D, Gao J (2015). Long non-coding rna expression profiling in aging rats with erectile dysfunction. Cellular Physiology and Biochemistry.

[ref-28] Podgrabinska S, Braun P, Velasco P, Kloos B, Pepper MS, Skobe M (2002). Molecular characterization of lymphatic endothelial cells. Proceedings of the National Academy of Sciences of the United States of America.

[ref-29] Ryu JK, Jin HR, Yin GN, Kwon MH, Song KM, Choi MJ, Park JM, Das ND, Kwon KD, Batbold D, Lee T, Gao ZL, Kim KW, Kim WJ, Suh JK (2013). Erectile dysfunction precedes other systemic vascular diseases due to incompetent cavernous endothelial cell–cell junctions. Journal d Urologie.

[ref-30] Saito R, Smoot ME, Ono K, Ruscheinski J, Wang PL, Lotia S, Pico AR, Bader GD, Ideker T (2012). A travel guide to Cytoscape plugins. Nature Methods.

[ref-31] Shamloul R, Ghanem H (2013). Erectile dysfunction. Lancet.

[ref-32] Szklarczyk D, Morris JH, Cook H, Kuhn M, Wyder S, Simonovic M, Santos A, Doncheva NT, Roth A, Bork P, Jensen LJ, Von Mering C (2017). The STRING database in 2017: quality-controlled protein–protein association networks, made broadly accessible. Nucleic Acids Research.

[ref-33] Thomas A, Woodard C, Rovner ES, Wein AJ (2003). Urologic complications of nonurologic medications. Urologic Clinics of North America.

[ref-34] Wessells H, Sullivan CJ, Tsubota Y, Engel KL, Kim B, Olson NE, Thorner D, Chitaley K (2009). Transcriptional profiling of human cavernosal endothelial cells reveals distinctive cell adhesion phenotype and role for claudin 11 in vascular barrier function. Physiological Genomics.

[ref-35] Zambon JP, Patel M, Hemal A, Badlani G, Andersson KE, Magalhaes RS, Lankford S, Dean A, Williams JK (2018). Nonhuman primate model of persistent erectile and urinary dysfunction following radical prostatectomy: feasibility of minimally invasive therapy. Neurourology and Urodynamics.

[ref-36] Zhang XR, Zhang ZJ, Zhu RX, Yuan YG, Jenkins TA, Reynolds GP (2011). Sexual dysfunction in male schizophrenia: influence of antipsychotic drugs, prolactin and polymorphisms of the dopamine D2 receptor genes. Pharmacogenomics.

